# Selecting Antidepressants Based on Medical History and Stress Mechanism

**DOI:** 10.7759/cureus.37117

**Published:** 2023-04-04

**Authors:** Hua Min, Farrokh Alemi, Janusz Wojtusiak

**Affiliations:** 1 Health Administration and Policy, George Mason University, Fairfax, USA

**Keywords:** individualized approach, medical history, stress mechanism, antidepressant, depression

## Abstract

Purpose

At present, clinicians typically prescribe antidepressants based on the widely accepted “serotonin hypothesis.” This study explores an alternative mechanism, the stress mechanism, for selecting antidepressants based on patients’ medical history.

Methods

This study investigated clinicians’ prescribing patterns for the 15 most common antidepressants, including amitriptyline, bupropion, citalopram, desvenlafaxine, doxepin, duloxetine, escitalopram, fluoxetine, mirtazapine, nortriptyline, paroxetine, ropinirole, sertraline, trazodone, and Venlafaxine. The least absolute shrinkage and selection operator (LASSO) logistic regression was used to identify factors that affect the remission of depression symptoms after receiving an antidepressant.

Results

The study found that a wide range of factors influenced the propensity of clinicians to prescribe antidepressants, with the number of predictors ranging from 51 to 206 variables. The prevalence of prescribing an antidepressant ranged from 0.5% for doxepin to 24% for the combination of more than one antidepressant. The area under the receiver operating curves (AROC) ranged from 77.2% for venlafaxine to 90.5% for ropinirole, with an average AROC of 82% for predicting the propensity of medications. A variety of diagnoses and prior medications affected remission, in agreement that the central mechanism for the impact of medications on the brain is through stress reduction. For example, psychotherapy, whether done individually or in a group, whether done for a short or long time, and whether done with evaluation/assessment or not, had an impact on remission. Specifically, teenagers and octogenarians were less likely to benefit from bupropion, citalopram, escitalopram, fluoxetine, and sertraline compared to patients between 40 and 65 years old. The findings of this study suggest that considering a patient’s medical history and individual characteristics is crucial for selecting the most effective antidepressant treatment.

Conclusions

Many studies have raised doubt about the serotonin hypothesis as the central mechanism for depression treatment. The identification of a wide range of predictors for prescribing antidepressants highlights the complexity of depression treatment and the need for individualized approaches that consider patients’ comorbidities and previous treatments. The significant impact of comorbidities on the response to treatment makes it improbable that the mechanism of action of antidepressants is solely based on the serotonin hypothesis. It is hard to explain how comorbidities lead to the depletion of serotonin. These findings open up a variety of courses of action for the clinical treatment of depression, each addressing a different source of chronic stress in the brain. Overall, this study contributes to a better understanding of depression treatment and provides valuable insights for clinicians in selecting antidepressants based on patients’ medical history.

## Introduction

Depression is treatable, but the mechanism of response to treatment is not yet fully understood. There are two types of treatment options available for depression: pharmacological and non-pharmacological. Non-pharmacological treatments include physical activity, cognitive behavior therapy (CBT), and general support [1,2]. Studies showed that exercise-based treatments for depression affected the brain, including the prefrontal cortex, anterior cingulate cortex, hippocampus, and corpus callosum [1]. Antidepressants are a popular pharmacological treatment choice for depression, and one of the most frequently prescribed medications in the USA. The major classes of antidepressants in practice include selective serotonin reuptake inhibitors (SSRIs), serotonin and norepinephrine reuptake inhibitors (SNRIs), norepinephrine and dopamine reuptake inhibitors (NDRIs), atypical antidepressants, tricyclic antidepressants, monoamine oxidase inhibitors (MAOIs), and other types of medications [3]. While both cognitive therapy and medications have been shown to be effective, the exact mechanism of action is still not clear.

In practice, clinicians prescribe antidepressants based on the “serotonin hypothesis.” According to this hypothesis, antidepressants inhibit the presynaptic reuptake of the neurotransmitter amines including noradrenaline, dopamine, and serotonin [4,5]. This hypothesis encourages clinicians to first prescribe antidepressants that work directly with serotonin reuptake inhibition; other medications are introduced if the first-order antidepressants fail [6]. Many studies have raised doubt about the serotonin hypothesis as the central mechanism for reducing depression and by extension raised concerns about the validity of two-tiered prescription guidelines [7-15]. First, patients’ responses to antidepressants are delayed significantly, suggesting a far more complex mechanism than a single serotonin pathway. Postsynaptic forebrain hyperactivity downstream of serotonin reuptake inhibitors changes the response to antidepressants, suggesting that more is at play than the serotonin hypothesis [16,17]. Second, our preliminary study has demonstrated that the response to antidepressants is mediated by over 400 comorbidities (e.g., anxiety state, abnormal weight gain, and unspecified tachycardia), procedures (e.g., family psychotherapy and psychiatric diagnostic evaluation), and current medications (e.g., allopurinol, fentanyl, and gabapentin) [18]. It is unlikely that these diverse sets of comorbidities all affect serotonin reuptake inhibitors. Third, data suggest that epigenetics, traumatic experiences, and environmental factors may affect responses to antidepressants, but the relationship between these factors and the serotonin hypothesis is not known or not credible [19]. Forth, the new research also suggests that in some patients, depressive behaviors may be fueled by the inflammatory process. Inflammation decreases the release of dopamine and reduces the functional connections between the ventral striatum and the prefrontal cortex, which are important parts of the brain’s reward circuitry [20]. Finally, functional magnetic resonance imaging and diffusion tensor imaging show that several brain regions, including the frontal lobe, parietal lobe, thalamus, caudate, pallidum, putamen, and temporal lobes, are significantly impaired in depressive patients [21], suggesting that there are multiple mechanisms for both depression and treatment. For these reasons, the field needs a more complex, nuanced, and, at the same time, more holistic picture of the mechanism of action of antidepressants.

Recent reviews on the effectiveness of antidepressants indicate that average differences among antidepressants are negligible [22-25], although large differences exist in response to antidepressants among subgroups of patients [18,26]. The confusion surrounding the prescription of different antidepressants has led to public media reports suggesting that “nobody can agree on the benefits of antidepressants” [27]. Prescribing antidepressants is challenging due to several reasons: (a) there are many available possible choices, with over 20 antidepressants currently on the market, which clinicians can combine with each other and with other medications; (b) the thousands of studies on the effectiveness of antidepressants, each relying on a small sample of patients, most exclude patients who present with multiple comorbidities [22,28] and severe depression [29]; (c) negative studies are not published and not available to practicing clinicians, although reported to the Food and Drug Administration (FDA) [30]; (d) genetic profiling has not been useful in determining responses to antidepressants [31]; and (e) few studies have clarified how patients’ comorbidities affect their response to antidepressants [22,28]. The growing difficulties in appropriately prescribing antidepressants have increased the need to understand why and when patients benefit from these antidepressants.

There is an alternative mechanism of action for antidepressants. We assumed that chronic stress, introduced by a variety of brain and body illnesses, affects stress, corticotropin-releasing factor (CRF) 1, endocrine, and other autonomic responses [18]. Brain and pituitary corticotropin-releasing factor receptors mediate endocrine, behavioral, and autonomic responses to stress [32]. Eventually, repeated stress affects the performance of the hypothalamic-pituitary-adrenal (HPA) axis [33,34]. Then, the activation of the HPA axis could be the key mechanism for the patient’s response to antidepressants [35-40]. This mechanism explains the role of chronic pain in depression [41], why chronic sleep deprivation increases depression [42] and exercise reduces mild depression [1,43], all of which affect stress levels. This opens up a wide variety of courses of action for the clinical treatment of depression, each addressing a different source of chronic stress.

A key assumption of the stress reduction mechanism is that patients’ comorbidities affect their responses to depression treatment. Several diagnoses are known to affect response to depression treatment, including cognitive disorders [12], substance use disorders [44,45], obesity [46,47], insomnia [48], cardiovascular or cerebrovascular diseases [49-51], hormone imbalances [52-54], cancer [55,56], and posttraumatic stress disorder [57]. However, a comprehensive list is not available. The purpose of this paper is to identify a more complete list of comorbidities that affect response to treatment and therefore further support the role of stress mechanism in the treatment of depression.

## Materials and methods

Source and size of data

The cohort was organized using claims data available through OptumLabs. The data included 71,721,417 patients during the timeframe between January 1, 2001, and December 31, 2018. Among these, 11,472,471 took one or more antidepressants and 6,897,748 also had a diagnosis of major depression. We excluded 2,790,721 patients who had a short medical history (defined as being eligible for the health plan for at least one year prior to their first antidepressant). After all inclusions and exclusions, we focused the analysis on 3,678,082 unique patients in 10,221,145 treatment episodes. The average follow-up period was 2.93 years, post their first antidepressant use. This cohort included a total of 15,096,055 person-years of data. Details of the selection of the cohort, including codes used to select the cohort, are available in Alemi et al. [26].

Measurement of remission

Patient-reported remission of depression symptoms was not consistently available in our data. Therefore, a surrogate measure was defined based on patterns of use of antidepressants, including (1) duration of use, (2) reaching therapeutic dose, (3) not switching from one antidepressant to another, (4) not augmenting the antidepressant with another medication, and (5) low use of antidepressants prior to the start of this medication [58].

Measurement of depression comorbidities

The study statistically controlled for patients’ history of illness-affected responses to antidepressants. Every diagnosis (i.e., International Classification of Diseases, Ninth Revision, Clinical Modification (ICD9CM)) is treated as a separate binary variable. A total of 16,811 outpatient predictors derived from the patients’ diagnoses were included in the analysis. For example, we statistically controlled for cognitive disorders, substance use disorders, obesity, diabetes, insomnia, cerebrovascular diseases, hormone imbalances, cancer, posttraumatic stress disorder, and other diseases [18].

Measurement of treatment of comorbidities

The study also statistically controlled for procedures or medications that affected responses to antidepressants. We defined a separate variable for each mental health procedure identified using either Current Procedural Terminology version 4 (CPT4) or ICD codes. A total of 4,364 binary procedure variables for mental health encounters were included in the analysis. We also included 4,253 medications as generic drug names, measured through the pharmacy claims data [10].

Methods of analysis

The least absolute shrinkage and selection operator (LASSO) logistic regression was used to identify factors that affect remission of depression symptoms after receiving one of the 15 most common antidepressants, including amitriptyline, bupropion, citalopram, desvenlafaxine, doxepin, duloxetine, escitalopram, fluoxetine, mirtazapine, nortriptyline, paroxetine, ropinirole, sertraline, trazodone, and venlafaxine. The area under the receiver operating curves (AROC) was used to measure the performance of regression models.

## Results

We observed that clinicians prescribed an antidepressant based on patients’ medical conditions including their comorbidities, procedures, and medication history. The number of robust variables for predicting the propensity of prescribing antidepressants ranged from 51 to 206 predictors (Table 1). The “Other” category included a combination of medications. The prevalence of prescribing an antidepressant ranged from 0.5% for doxepin to 24% for Other (e.g., combination). The AROC ranged from 77.2% for venlafaxine to 90.5% for ropinirole. The average AROC for predicting the propensity of medications was 82%. These data suggest that the regression equations explained a large portion of the variance in response to treatment.

**Table 1:** Cross-validated accuracy of LASSO logistic models Separate models were conducted for each medication. Other categories included the combination of medications. LASSO: least absolute shrinkage and selection operator, AROC: area under the receiver operating curve

Antidepressant	Prescribing the antidepressant		
	Prevalence	AROC	# predictors
Amitriptyline	2.8%	85%	134
Bupropion	8.9%	77.9%	156
Citalopram	9.1%	81%	168
Desvenlafaxine	0.8%	82.3%	83
Doxepin	0.5%	84.1%	51
Duloxetine	4.4%	79.7%	118
Escitalopram	10.6%	79%	155
Fluoxetine	8.7%	82.9%	95
Mirtazapine	1.8%	84.6%	124
Nortriptyline	0.9%	84%	62
Other	24%	77.4%	206
Paroxetine	4.7%	84.9%	136
Ropinirole	0.6%	90.5%	55
Sertraline	12.3%	81.9%	122
Trazodone	4.6%	79.3%	105
Venlafaxine	5.3%	77.2%	72

Table 2 summarizes the important predictors, presented as ICD9CM and CPT4 codes, for patients in each subgroup of age and gender who had received those 15 antidepressants. As shown in Table 2, age groups 20-40 and 41-64 had the most complex medical history with many distinct ICD9CM and CPT4 codes when compared to other age groups. Some codes commonly existed in the majority of the strata such as depressive disorder, not elsewhere classified (311), anxiety state, unspecified (300.XX), major depressive affective disorder, recurrent episode, unspecified (296.30), and psychotherapy (90801 and 90805-90807). Comorbidity codes started in the age group of 20-40, including diabetes mellitus (250.00) and neoplasm of uncertain behavior of the skin (238.2). Some codes were unique for certain age groups and gender. For example, normal pregnancy (V22.XX) appeared among women in the age group of 20-40 only. Some codes appeared in certain antidepressants such as chronic airway obstruction, not elsewhere classified (496) in the bupropion. The most common procedure codes were psychotherapy codes including psychiatric diagnostic interview examination (90801) and pharmacologic management (90862).

**Table 2:** ICD9CM and CPT4 codes that affect response to antidepressants ICD9CM: International Classification of Diseases, Ninth Revision, Clinical Modification, CPT4: Current Procedural Terminology version 4

Age: 13-19 | gender: female	Age: 13-19 | gender: male	Age: 20-40 | gender: female	Age: 20-40 | gender: male	Age: 41-64 | gender: female	Age: 41-64 | gender: male	Age: 65-79 | gender: female	Age: 65-79 | gender: male	Age: 80-89 | gender: female	Age: 80-89 | gender: male
ICD9: 311, 34690, 7840, V202, V7231 CPT4: 90801	ICD9: 34690, 7840, V202	ICD9: 29630, 30000, 30002, 3004, 311, 34690, 5641, 6254, 6259, 7291, 78052, 7840, V221, V270, V723, V7231, V7612 CPT4: 90801, 90805, 90862	ICD9: 29630, 30002, 311, 34690, 5641, 7291, 78052, 7840 CPT4: 90862	ICD9: 0539, 25001, 2722, 29630, 29632, 30000, 30002, 3004, 311, 32723, 34690, 3559, 3572, 3569, 5641, 6254, 6259, 7291, 78052, 78079, 7820, 7840, 78900, V723, V7231, V7612 CPT4: 90801, 90805, 90862	ICD9: 0539, 25001, 2722, 29630, 30000, 30002, 3004, 311, 32723, 34690, 3569, 3572, 5641, 7242, 7291, 78052,7820, 7840, V700 CPT4: 90801, 90862	ICD9: 0539, 25001, 29630, 30002, 311, 32723, 3569, 3572, 7291,78052, 7840, 7231, V7612	ICD9: 311, 32723, 3569, 3572, 7840	ICD9: 311, 7840, V7612	
ICD9: 29620, 29622, 29630, 29635, 30000, 30002, 3051, 30928, 311, 31400, 31401, 3671, 78079, 7831, 7840 CPT4: 90801, 90805, 90862	ICD9: 29620, 29622, 29630, 29635, 30000, 30002, 3051, 30928, 311, 31400, 31401, 3671, 78079, V700 CPT4: 90801, 90805, 90862	ICD9: 2382, 25000, 2564, 2599, 27800, 27801, 27802, 29620, 29622, 29630, 29635, 29636, 29689, 29690, 30000, 30001, 30002, 3003, 3051, 3090, 30928, 311, 31400, 31401, 34690, 3671, 496, 5641, 6254, 70400, 7291, 78039, 78052, 78079, 7831, 78321, 7840, 78900, V016, V221, V222, V270, V698, V723, V700, V7231 CPT4: 90801, 90805, 90806, 90807, 90862	ICD9: 2382, 25000, 27800,27801,29620, 29622,29630,29635, 29689, 29690, 30000, 30001, 30002, 3003, 30390, 3051, 3090, 30928, 311, 31400, 31401, 3671, 4019, 60784, 7245, 7291, 78039, 78052, 78079, 7831, 78321, 7840, 78900, V5869, V698, V700 CPT4: 90801, 90805, 90806, 90862	ICD9: 1749, 2382, 25000, 2564, 2599, 2777, 27800, 27801, 27802, 2859, 29620, 29621, 29622, 29630, 29635, 29636, 29689, 29690, 30000, 30001, 30002, 3003, 3051, 3090, 30928, 311, 31400, 31401, 33394, 3384, 34590, 34690, 3569, 3671, 4011, 4019, 4280, 436, 496, 5641, 6254, 6272, 70400, 7140, 71590, 7291, 73300, 78039, 78052, 78079, 7831, 78321, 7840, 78900, V221, V5869, V5881, V698, V700, V723, V7231 CPT4: 90801, 90805, 90806, 90807, 90862	ICD9: 2382, 25000, 2572, 2777, 27800, 27801, 29620, 29622, 29630, 29635, 29636, 29689, 29690, 30000, 30001, 30002, 3003, 30390, 3051, 3090, 30928, 311, 31400, 31401, 3671, 4011, 4019, 4280, 436, 496, 60784, 7291, 78039, 78052, 78079, 7831, 78321, 7840, V5869, V5881, V698, V700 CPT4: 90801, 90805, 90806, 90807, 90862	ICD9: 1749, 25000, 27800, 29630, 29635, 29636, 30000, 30002, 3051, 311, 3671, 496, 78079, 7831, V698, V700	ICD9: 25000, 27800, 29630, 29635, 29636, 30000, 3051, 311, 496, 60784, 78079	ICD9: 29630, 496	ICD9: 29630
ICD9: 29630, 30928, 311, 3671, 4619, 61610, 7061, V016, V202, V6284, V723, V745 CPT4: 90791, 90801, 90805, 90806, 90807, 90834, 90847, 90862	ICD9: 30928, 311, 7061, V202 CPT4: 90791, 90801, 90805, 90806, 90807, 90834, 90847, 90862	ICD9: 1101, 29630, 29632, 29635, 29680, 30000, 30001, 3004, 3090, 30928, 30981, 311, 32723, 34610, 34690, 3671, 4011, 4619, 4659, 3500, 56400, 5990, 5997, 61610, 6235, 6238, 6254, 6259, 6260, 6264, 69010, 7061, 7291, 7295, 7804, 78052, 7806, 78071, 78079, 78099, 78321, 7840, 7851, 78650, 78703, 78900, 9953, 9999, V016, V202, V221, V222, V242, V2509, V5889, V6284, V698, V720, V723, V726, V7260, V7283, V745, V762, V8289, V829, V851 CPT4: 90791, 90792, 90801, 90805, 90806, 90807, 90833, 90834, 90837, 90847, 90862	ICD9: 1101, 2572, 29630, 29632, 29635, 30000, 30001, 3004, 30390, 30500, 3051, 30928, 311, 32723, 3671, 4011, 4619, 4659, 7061, 7244, 7291, 7295, 78052, 7806, 78079, 78321, 7840, 7851, 78650, 78703, 78900, V016, V6284, V698, V720, V726, V745 CPT4: 90791, 90792, 90801, 90805, 90806, 90807, 90833, 90834, 90862	ICD9: 1101,29630,29632,29635, 29680, 30000, 30001,30742, 3090, 30928, 30981, 311, 32723, 33394, 33399, 33829, 3384, 34610, 34690, 3579, 3671, 37300, 37515, 3899, 4011, 40210, 4619, 53500, 5533, 56400, 5990, 5997, 59970, 6101, 61610, 6238, 6254, 6259, 6264, 6272, 69010, 70219, 7061, 70909, 7244, 7291, 7295, 73399, 7804, 78052, 7806, 78071, 7809, 78321, 7840, 7851, 78650, 7872, 78900, 79319, 9953, 9999, V016, V5869, V5889, V698, V720, V723, V726, V7260, V7283, V745, V7612, V762, V8289, V829, V851 CPT4: 90791, 90792, 90801, 90805, 90806, 90807, 90833, 90834, 90837, 90847, 90862	ICD9: 1101, 2572, 2724, 29630, 29632, 29635, 30000, 30390, 30500, 3090, 30928, 311, 32723, 33394, 3384, 3671, 4011, 40210, 4619, 5533, 56400, 5997, 7244, 7291, 78052, 7806, 78071, 78321, 7851, 78650, 79319, V016, V5869, V5889, V698, V720, V726, V7283, V745, V7644 CPT4: 90791, 90792, 90801, 90805, 90806, 90807, 90833, 90834, 90837, 90862	ICD9: 1101, 29010, 2948, 29630, 29632, 29635, 3090, 311, 32723, 3320, 33394, 3579, 3671, 37300, 37515, 4011, 40210, 44020, 5533, 56400, 5997, 61610, 7291, 73399, 78052, 78071, 78321, 7851, 79319, V5889, V698, V7283, V8289, V851 CPT4: 90792, 90792, 90801, 90806, 90834, 90862	ICD9: 1101, 29010, 2948, 29630, 29635, 311, 32723, 3320, 4011, 44020, 78052, 78321, 7851, 79319, V5889, V698, V7644 CPT4: 90791, 90801, 90806, 90834, 90862	ICD9: 1101, 29010, 2948, 29630, 29635, 311, 32723, 3320, 4011, 44020, 78321, 79319, V5889, V851 CPT4: 90791, 90792, 90801, 90834	ICD9: 1101, 29010, 32723, 3320, 4011, 44020, 78321
CPT4: 90833, 90834		ICD9: 29630,29635, 311, 78079, V7231 CPT4: 90801, 90805, 90806, 90807, 90833, 90834, 90862	ICD9: 311, 29630, 78079 CPT4: 90801, 90805, 90806, 90807, 90833, 90834, 90862	ICD9: 29630, 29635, 311, 78079, V7231 CPT4: 90801, 90805, 90806, 90807, 90833, 90834, 90862	ICD9: 29630, 311 CPT4: 78079, 90801, 90805, 90806, 90807, 90833, 90834, 90862	ICD9: 29630, 311			
		ICD9: 29630, 311, 6929, 7089, V270, V7231 CPT4: 90806, 90835		ICD9: 2689, 29630, 30742, 311, 34690, 38181, 6918, 6929, 6989, 7089, 78052, 7821, V700, V7231 CPT4:90806, 90834	ICD9: 29630, 311, 6929, 7089, 78052, V700, CPT4: 90806, 90834	ICD9: 6929, 311, 4011, 78052, 6989, 7089			
ICD9: 29630, 7291 CPT4: 90806, 90834, 90862	CPT4: 90862	ICD9: 30928, 29630, 29635, 311, 61610, 6238, 71940, 71949, 7291, 78079, 7851, V221, V222, V723, V7231, V745 CPT4: 90791, 90801, 90805, 90806, 90807, 90833, 90834, 90836, 90862	ICD9: 29630, 29635, 311, 7291, 78079 CPT4: 90801, 90805, 90806, 90807, 90834, 90862	ICD9: 2777, 29620, 29630,29632, 29635, 30928, 311, 33394, 340, 3559, 3569, 4011, 61610, 6238, 7140, 71940, 71949, 7242,7291, 78079,7851, V6549, V723, V7231, V745, V851 CPT4: 90801, 90833, 90836, 90791	ICD9: 29630, 29635, 311, 3569, 4011, 71940, 71949, 7242, 7291, 78079, 7851, V851 CPT4: 90791, 90801, 90805, 90806, 90807, 90833, 90834, 90862	ICD9: 29630, 29635, 311, 3310, 3569, 3579, 4011, 7140, 71940, 71949, 7291, 78079, 7851, V7231, V851 CPT4: 90801, 90806, 90834, 90862	ICD9: 29630, 29635, 311, 3569, 4011, 7291, 78079 CPT4: 90801, 90834	ICD9: 29630, 29635, 3310, 7291, 78079	ICD9: 7291
ICD9: 29630, 29635, 30000, 30002, 3051, 3090, 30928, 311, 34690, 3671, 5990, 61610, 6254, 6260, 7291, 78079, 7851, 78650, 9999, V700, V723, V7231 CPT4: 90801, 90805, 90806, 90807, 90833, 90834, 90836, 90837, 90862	ICD9: 29630, 30000, 30928, 311, V700 CPT4: 90791, 90792, 90801, 90805, 90806, 90807, 90833, 90834, 90837, 90862	ICD9: 1101, 2165, 27801, 29630, 29631, 29633, 29635, 30000, 30002, 3003, 3051, 311, 3089, 3090, 30924, 30928, 32723, 340, 34690, 56400, 3671, 5990, 61171, 61610, 6254, 6260, 7291, 7295, 7804, 78079, 7831, 7840, 7851, 78650, 7873, 78900, 79500, 9999, V5869, V700, V723, V7231, V726, V745, V851 CPT4: 90791, 90792, 90801, 90805, 90806, 90807, 90833, 90834, 90836, 90837, 90862	ICD9: 1101, 2165,2572, 27801, 29630, 29635 30000, 30002, 3003, 3051, 3089, 3090, 30928, 311, 32723, 34690, 3671, 7242, 7291, 7295, 7804, 78052, 78079, 7851, 78650, 78900, 9999, V5869, V700, V745 CPT4: 90791, 90792, 90801, 90805, 90806, 90807, 90833, 90834, 90836, 90837, 90862	ICD9: 1101, 2165, 27801, 29630, 29631, 29635, 30000, 30002, 3003, 3051, 3089, 3090, 30924, 30928, 311, 32723, 33394, 33399, 340, 34690, 3671, 3674, 4240, 43491, 4439, 56400, 5990, 61171, 61610, 6254, 6260, 7092, 7291, 7295, 7804, 78052, 78079, 7831, 7851, 78650, 7873, 79500, 9999, V5869, V700, V723, V7231, V851 CPT4: 90791, 90792, 90801, 90805, 90806, 90807, 90833, 90834, 90836, 90837, 90862	ICD9: 1101, 2165, 2572, 27801, 29630, 29635, 30000, 30002, 30390, 3051, 3089, 3090, 30928, 311, 32723, 33394, 340, 34690, 3671, 4240, 43491 4439, 7291, 78052, 78079, 7851, 78650, 9999, V5869, V700, CPT4: 90791, 90792, 90801, 90805, 90806, 90807, 90833, 90834, 90836, 90837, 90862	ICD9: 1101, 27801, 29010, 29630, 29635, 30000, 30002, 3051, 3090, 30928, 311, 32723, 3320, 33394, 4240, 43491, 4439, 7291, 7851, 78650, V5869, V723, V7231 CPT4: 90791, 90801, 90806, 90834			
ICD9: 29630, 29635, 30000, 3090, 30928, 311, 3671, 4659, 5990, 61610, 6254, 7291, 7804, 78052, 78079, 78321, 7840, 7851, 78703, 78900, V016, V202, V221, V2509, V723, V7231 CPT4: 90791, 90792, 90801, 90805, 90806, 90807, 90833, 90834, 90836, 90837, 90847, 90862	ICD9: 29630, 30928, 311, 3671, 4659, V202 CPT4: 90791, 90792, 90801, 90805, 90806, 90807, 90833, 90834, 90836, 90862	ICD9: 1101, 1104, 1121, 2113, 2189, 27800, 29622, 29630, 29632, 29633, 29635, 29680, 30000, 30001, 30002, 3004, 30500, 3051, 3090, 30928, 311, 32723, 340, 34690, 3671, 37515, 4659, 56400, 5990, 6101, 61171, 61610, 6254, 6259, 70400, 7068, 70909, 7231, 7291, 7295, 7804, 78052, 78079, 78321, 7840, 7851, 78650, 78703, 78900, V016, V202, V221, V2509, V5869, V723, V7231 CPT4: 90791, 90792, 90801, 90805, 90806, 90807, 90833, 90834, 90836, 90837, 90862	ICD9: 29630, 29632, 29635, 30000, 30002, 3004, 30500, 3051, 30928, 311, 32723, 3671, 4659, 7242, 7291, 7804, 78052, 78079, 78321, 7840, 7851, 78650,78900, V016, V5869 CPT4: 90791, 90792, 90801, 90805, 90806, 90807, 90833, 90834, 90836, 90862	ICD9: 1101, 1104, 1121, 1749, 2113, 2189, 2722, 2724, 27800, 29622, 29630, 29632, 29633, 29635, 29680, 30000, 30001, 30002, 3004, 30500, 3051, 3090, 30928, 311, 32723, 33394, 33399, 340, 34690, 3671, 37515, 4011, 4019, 4280, 4359, 4659, 56400, 5990, 6101, 61171, 61610, 6254, 6259, 70400, 7068, 70909, 7231, 7242, 7291,7295, 73300, 7804, 78052, 78079, 78321, 7840, 7851, 78650, 78900, V016, V221, V5869, V723, V7231 CPT4: 90791, 90792, 90801, 90805, 90806, 90807, 90833, 90834, 90836, 90837, 90862	ICD9: 1101, 1104, 2113, 2724, 29630, 29632, 29635, 30000, 30002, 3004, 30500, 3051, 30928, 311, 32723, 3671, 4011, 4019, 4280, 4359, 4659, 7291, 7804, 78052, 78079, 78321, 7840, 7851, 78650, V5869 CPT4: 90791, 90792, 90801, 90805, 90806, 90807, 90833, 90834, 90862	ICD9: 1101, 1749, 2113, 29630, 29632, 29635, 3310, 311, 33394, 37515, 4280, 4359, 44020, 7291, 73300, 78052, 78321, 7851 CPT4: 90801, 90806, 90834, 90862	ICD9: 1101, 2113, 29630, 29635, 311, 3310, 4280, 4359, 44020, 78052	ICD9: 1101, 29630, 3310	
	ICD9: 31401	ICD9: 29630, 78052, 78321, V270, V7231 CPT4: 90801	ICD9: 29630, 3051, 4619, 31401, 78052, 78321 CPT4: 90801, 90834, 90899	ICD9: 27800, 27801, 29630, 29635, 3051, 4019, 4619, 73300, 78052, 7830, 7831, 78321, V7231 CPT4: 90801, 90834, 90862	ICD9: 1101, 27800, 29630, 29635, 3051, 30928, 32723, 4019, 78052, 78321, V7644 CPT4: 90801, 90862, 90834	ICD9: 1101, 2689, 27800, 27801, 2948, 29630, 29635, 3051, 32723, 36511, 4019, 73300, 73399, 78052, 7830, 78321, V7231 CPT4: 90801, 90862	ICD9: 1101, 27800, 29630, 3051, 32723, 36511, 4019, 78052, 7830, 78321, V7644 CPT4: 90801	ICD9: 1101, 3310, 7830, 78321	
ICD9: 7840	ICD9: 7840	ICD9: 29630, 30000, 311, 34610, 34690, 5641, 7291, 7804, 7820, 7840, 78900, V221 CPT4: 90801, 90834	ICD9: 7840, 34690	ICD9: 29630, 29635, 30000, 311, 34610, 34690, 3569, 5641, 7231, 7244, 7291, 7295, 7804, 7820, 7840, 78900, 8470 CPT4: 90801, 90834	ICD9: 29630, 311, 34690, 3569, 7244, 7820, 7840, 78900	ICD9: 29630, 30000, 311, 3569, 4400, 5641, 7840	ICD9: 3569, 7840	ICD9: 7840	
ICD9: 3000, 311, V202, V723, V7231 CPT4: 90801, 90806, 90834, 90862	ICD9: V202 CPT4: 90801, 90806, 90862	ICD9: 29630, 30000, 30001, 30928, 311, 3671, 61610, 6254, 625, 7291,7336, 7392, 78079, 7831, 78321, 7851, V202, V221, V700, V723, V7231, V726, V745 CPT4: 90791, 90801, 90805, 90806, 90807, 90834, 90862	ICD9: 2572, 29630, 30000, 30001, 30928, 311, 31400, 32723, 3671, 4659, 7291, 7295, 7392, 78052, 7806, 78079, 7831, 78321, 7851, 78650, V700, V726, V7791 CPT4: 90791, 90801, 90805, 90806, 90807, 90834, 90862	ICD9: 1101, 2189, 29630, 30000, 30001, 30928, 311, 32723, 3671, 4011, 53500, 56400, 5997, 61610, 6254, 6272, 7291, 7295, 7336, 7392, 78052, 7806, 78079, 7831, 78321, 7851, 78650, 7873, V700, V723, V7231, V726, V7611, V7791 CPT4: 90791, 90801, 90805, 90806, 90807, 90834, 90862	ICD9: 1101, 25000, 2572, 29630, 30000, 30001, 30928, 311, 32723, 4011, 5997, 7291, 7392, 78052, 78079, 7831, 78321, 7851, 78650, V700, V726, V7791 CPT4: 90791, 90801, 90805, 90806, 90807, 90834, 90862	ICD9: 1101, 29630, 30000, 30001, 311, 32723, 3310, 4011, 7291, 7831, 78321, 7851, 7873, V700, V723, V7231 CPT4: 90801, 90806, 90834, 90862	ICD9: 1101, 2572, 29630, 30000, 311, 32723, 3310, 3320, 60001, 78321, 7851	ICD9: 1101, 29630, 30000, 311, 3310	
		ICD9: 33399, 33394	ICD9: 33399, 33394	ICD9: 25000, 2689, 30000, 311, 32723, 3320, 33394, 33399, 78053	ICD9: 311, 32723, 3320, 33394, 33399, 78053	ICD9: 25000,2689, 30000, 311, 3320, 33394, 33399	ICD9: 25000, 32723, 3320, 33394, 33399	ICD9: 33394	
ICD9: 07999,29622, 29623, 29630, 29632, 29635, 30000, 30001, 3090, 30924, 30928, 30981, 311, 3671, 4619, 56400, 5990, 61610, 6259, 6260, 7291, 78052, 78079, 7831, 78321, 7840, 78650, 78900, V016, V202, V221, V222, V6284, V700, V723, V745 CPT4: 90791, 90792, 90801, 90805, 90806, 90807, 90833, 90834, 90836, 90837, 90847, 90862	ICD9: 07999, 29630, 30928 311, 3671, 4619, 7291, 78052, 78900, V202, V6284, V700 CPT4: 90806, 90791, 90792, 90801, 90805, 90807, 90834, 90837, 90847, 90862	ICD9: 07999,2189,2689, 27802, 29620, 29622, 29623, 29630, 29632, 29633, 29635, 29680, 30000, 30001, 30002, 3004, 3051, 30742, 3090, 30924, 30928, 30981, 3099, 311, 32723, 340, 34610, 34611, 34690, 3671, 38870, 4619, 56400, 5990, 61171, 61610, 6259, 6260, 6264, 65963, 66411, 7291, 7295, 7391, 7804, 78052, 78071, 78079, 7831, 78321, 7840, 7851, 78650, 78659, 7873, 78900, V016, V202, V221, V222, V2541, V283, V286, V288, V700, V723, V7260, V745, V7612, V762, CPT4: 90806, 90791, 90792, 90801, 90805, 90807,90833, 90834, 90836, 90837,90847, 90862	ICD9: 07999, 2572, 29620, 29622, 29630, 29632, 29635, 30000, 30001, 30002, 3004, 30390, 3051, 3090, 30928, 311, 32723, 34690, 3671, 4011, 4619, 56400, 5990, 60784, 7291, 7295, 7391,7804,78052, 78079, 7831, 8321, 7840, 7851, 78650, 78659, 7873, 78900, V016, V202, V6284, V700, V745 CPT4: 90806, 90791, 90792, 90801, 90805, 90807, 90833, 90834, 90836, 90837, 90862	ICD9: 07999, 1749, 2189, 2689, 2724, 27801, 27802, 29620, 29622, 29630,29632,29633,29635, 29680, 30000, 30001, 30002, 3004, 30390, 3051, 30742, 3090, 30924, 30928, 30981,3099, 311, 32723, 33394, 33399, 340, 34610, 34611, 34690, 3559, 3569, 36511, 3671, 37515, 4011, 4556, 45981, 4619, 56400, 5990, 61171, 61610, 6259, 6260, 6272, 65963, 7020, 71949, 7291, 7391, 7804, 78052, 78071, 78079, 7831, 78321, 7840, 7851, 78650, 78659, 7873, 78900, V016, V221, V222, V5881, V700, V723, V7260, V745, V7612, V762 CPT4: 90791, 90792, 90801, 90805, 90806, 90807, 90833, 90834, 90836, 90837, 90847, 90862	ICD9: 07999, 2572, 2689, 2724, 29622, 29630, 29632, 29635, 29680, 30000, 30001, 30002, 30390, 3051, 30742, 3090, 30928, 30981, 311, 32723, 33394, 3569, 3671, 4011, 4619, 56400, 5990, 60784, 7020, 7291, 7804, 78052, 78053, 78079, 7831, 78321, 7851, 78650, 78659, 78900, V016, V5881, V700, V7644 CPT4: 90791, 90792, 90801, 90805, 90806, 90807, 90833, 90834, 90836, 90837, 90862	ICD9: 1749, 2689, 29630, 29632, 29635, 3051, 30742, 3090, 30928, 311, 32723, 3320, 33394, 3569, 36511, 3671, 4011, 44020, 56400, 5990, 61171, 61610, 6259, 7020, 7291, 78052, 7831, 78321, 7851, 78650, V5881, V7612 CPT4: 90791, 90792, 90801, 90806, 90833, 90834, 90862	ICD9: 2572, 29630, 29632, 29635, 3051, 311, 32723, 3320, 3569, 36511, 56400, 60784, 7291, 78052, 78321, 78650, V5881, V7644 CPT4: 90791, 90801, 90806, 90834, 90862	ICD9: 1749, 2689, 29630, 29635, 311, 32723, 3320, 36511, 44020, 56400, 5990, 7291, 78052, 78321, 78650 CPT4: 90791, 90792, 90801, 90834	ICD9: 29630, 78052
ICD9: 29630, 311, 78052, V202 CPT4: 90805, 90806, 90834, 90862	ICD9: 29630, 311, 78052, V202 CPT4: 90805, 90806, 90834, 90862	ICD9: 29630, 29635, 30000, 3004, 30400, 3051, 311, 31401, 6254, 7291, 78050, 78052, 78079, V221, V270, V700 CPT4: 90801, 90805, 90806, 90807, 90833, 90834, 90862	ICD9: 29630, 29635, 30000, 30400, 30500, 3051, 30742, 311, 7291, 78050, 78052, V700 CPT4: 90801, 90805, 90806, 90807, 90833, 90834, 90862, 90899	ICD9: 29620, 29630, 29631, 29632, 29635, 30000, 3004, 30400, 30500, 3051, 30742, 311, 4019, 4400, 6254, 71941, 7242, 7291, 78050, 78052, 78079, V4364, V700, V7651 CPT4: 90801, 90805, 90806, 90807, 90833, 90834, 90862	ICD9: 29630, 29635, 2989, 30000, 3004, 30390, 30400, 30500, 3051, 30742, 311, 32723, 4019, 4400, 7291, 78050, 78052, 78079, V4364, V700, V7651 CPT4: 90801, 90805, 90806, 90807, 90833, 90834, 90862, 90899	ICD9: 29630, 29635, 30000, 3004, 3051, 30742, 311, 32723, 3320, 4019, 4400, 78050, 78052, 78079, V4364, V700, V7651, V851 CPT4: 90833, 90834, 90862	ICD9: 29630 29635, 30000, 3051, 30742, 311, 32723, 3320, 4019, 4400, 78050, 78052, V700, V7651 CPT4: 90834 90862	ICD9: 29630, 29635, 30000, 30742, 311, 4400, 78052, 78079, V4364, V700	ICD9: 29630, 311, 4400, 78052
ICD9: 29630, 311, V202, V723 CPT4: 90801, 90805, 90806, 90807, 90834, 90862, 90862	ICD9: 29630, 311, V202 CPT4: 90801, 90805, 90806, 90834, 90862	ICD9: 1749, 2189, 2689, 29620, 29630, 29632, 29635, 30000, 3004, 30928, 311, 4011, 61171, 61610, 6238, 6272, 7291, 7295, 7804, 78060, 78079, 7831, 78321, 78659, 78900, 9999, V016, V221, V723, V7381, V745, V762 CPT4: 90791, 90801, 90805, 90806, 90807, 90833, 90834, 90862	ICD9: 29620, 29630, 29635, 30000, 3004, 30928, 311, 4011, 78079, 78900 CPT4: 90791, 90801, 90805, 90806, 90807, 90833, 90834, 90862	ICD9: 1749, 2189, 2689, 29620, 29630, 29632, 29635, 30000, 3004, 30928, 311, 32723, 33394, 33829, 4011, 4019, 42789, 4738, 4780, 61171, 61172, 61610, 6238, 6272, 70400, 72887, 7291, 7295, 73300, 7804, 78060, 78079, 7831, 78321, 78659, 78720, 78900, 79380, 79389, 9999, V723, V7260, V7381, V745, V762 CPT4: 90791, 90801, 90805, 90806, 90807, 90833, 90834, 90837, 90862	ICD9: 2689, 29620, 29630, 29635, 30000, 3004, 30928, 311, 32723, 33829, 4011, 4019, 42789, 7291, 78079, 78321, 78659, 78900, 9999 CPT4: 90791, 90801, 90805, 90806, 90807, 90833, 90834, 90862	ICD9: 1749, 2689, 29630, 29635, 311, 4011, 6272, 72887 CPT4: 90801, 90805, 90806, 90833, 90834, 90862	ICD9: 29630, 29635, 311, 4011 CPT4: 90834, 90862	ICD9: 1749, 29630, 311	

Table 3 provides a partial list of robust predictors of remission for the top 5 most common antidepressants. A complete list is available in the Appendices. An examination of the coefficients of these predictors shows that psychotherapy, whether done individually or in a group, whether done for a short or long time, and whether done with evaluation/assessment or not, had an impact on remission. If the patient had a history of participating in psychotherapy and depression had returned, they were unlikely to benefit from citalopram, escitalopram, fluoxetine, or sertraline, but they could benefit from bupropion. The same was also true if the patient had gone through a session for pharmacological management or psychiatric evaluations: they would benefit from bupropion, but not the other remaining four common antidepressants. Teenagers and octogenarians were less likely to benefit from these five antidepressants than 40- to 65-year-old patients, although considerable variation existed. 

**Table 3:** Robust predictors of remission for the top 5 most common antidepressants

Significant and robust independent variables in one-year medical history	Response variable: remission				
	Patients on bupropion	Patients on citalopram	Patients on escitalopram	Patients on fluoxetine	Patients on sertraline
Family psychotherapy, 50 minutes		-0.17		-0.09	-0.14
Individual psychotherapy, 20-30 minutes	0.18	-0.57	-0.25	-0.53	-0.58
Individual psychotherapy, 45-50 minutes	0.09	-0.60	-0.31	-0.55	-0.62
Psychotherapy, 30 minutes with patient			-0.18	-0.21	-0.27
Psychotherapy, 30 minutes with an evaluation and management service		-0.72	-0.37	-0.58	-0.61
Psychotherapy, 45 minutes with patient		-0.64	-0.37	-0.55	-0.63
Psychotherapy, 45 minutes with an evaluation and management service			-0.43	-0.64	-0.79
Psychotherapy, 60 minutes with patient		-0.28	-0.19	-0.28	-0.35
Pharmacologic management	0.33	-0.60	-0.19	-0.58	-0.60
Psychiatric diagnostic evaluation		-0.28	-0.18	-0.28	-0.30
Psychiatric diagnostic evaluation with medical services		-0.26	-0.16	-0.19	-0.17
Psychiatric diagnostic interview examination	0.13	-0.43	-0.23	-0.38	-0.47
Age: 13-19		-0.53	-0.19	-0.54	-0.48
Age: 20-40	-0.10	-0.17	-0.08	-0.18	-0.16
Age: 65-79			-0.13	0.10	0.03
Age: 80-89		-0.35	-0.43	-0.24	-0.24
Gender: female	-0.07		-0.02	0.04	0.03
Last antidepressant different and no remission	-0.54	-0.18	-0.26	-0.19	-0.19
Last antidepressant different and remission	0.77	0.37	0.61	0.41	0.36
Last antidepressant same and no remission	-0.84	-0.91	-0.99	-0.90	-0.92
Last antidepressant same and remission	1.91	0.29	1.33	0.24	0.21
2 or 3 previous antidepressants			0.09		
4+ previous antidepressants			-0.27	-0.15	
2+ previous episodes	-0.11	-0.20	-0.16	-0.16	-0.18
2+ previous remissions	0.44	0.40	0.52	0.44	0.47
Buspirone			0.14		
Citalopram hydrobromide		0.73	0.45	0.15	0.14
Desogestrel-ethinyl estradiol					0.11
Duloxetine		0.22	0.28	0.16	0.19
Escitalopram oxalate		0.55	0.94	0.2	0.19
Estrogen, con/m-progest acet		0.14		0.15	0.15
Estrogen, ester/me-testosterone				0.11	
Fenofibrate		0.11		0.12	0.18
Fentanyl			0.13		
Fexofenadine/pseudoephedrine		0.11			
Fluoxetine		0.2	0.23	0.66	0.18
Hydroxychloroquine sulfate					0.12
Methylphenidate	0.16				
Modafinil	0.23				
Norethindrone					0.13
Oxycodone			0.11		
Paroxetine		0.2	0.22	0.12	0.18
Ramipril					0.12
Varenicline tartrate		0.12			
Venlafaxine		0.2	0.24	0.13	0.2
Elderly multigravida, with antepartum condition or complication					0.12
Fitting and adjustment of vascular catheter					0.12
Gynecological examination		0.13	-0.21		0.09
Major depressive disorder, recurrent episode, in full remission	2.08				
Major depressive disorder, recurrent episode, in partial or unspecified remission	2.14	1.31	1.51	1.37	1.31
Major depressive disorder, recurrent episode, mild			0.12		
Major depressive disorder, recurrent episode, unspecified	2.02	0.90	1.16	0.97	0.87
Major depressive disorder, single episode, moderate	0.15	-0.08		-0.06	-0.12
Major depressive disorder, single episode, unspecified	0.16				
Morbid obesity			0.13	0.05	0.09
Multiple sclerosis			0.13	0.17	0.10
Myopia	0.15	0.09		0.08	0.08
Obstructive sleep apnea		0.15	0.17	0.07	0.11
Routine postpartum follow-up		0.15			
Supervision of other normal pregnancy		0.12		0.12	0.10

## Discussion

Depression is heterogeneous. There are 227 possible ways to meet the symptom criteria for major depressive disorder [59]. For many people, this makes it difficult to find an effective treatment since there is no one-size-fits-all approach. Clinicians prescribe antidepressants based on the “serotonin hypothesis,” but only about 40% of patients have an improvement in their symptoms following the first treatment [60]. Thus, the majority of patients do not benefit from their first treatment, typically an SSRI. The serotonin hypothesis is a part of the monoamine hypothesis, which also includes the roles of dopamine and norepinephrine. Additionally, factors such as neuroinflammation, circadian rhythms, genes, environment, and neurotrophic factors are known to play roles in depression. The role of comorbidities in the management of depression has been known, but it has not always been clear by what mechanism comorbidities affect depression. In other diseases, comorbidities worsen the prognosis of patients by increasing stress on vital functions. The significant impact of comorbidities on the response to treatment makes it improbable that the mechanism of action of antidepressants is solely based on the serotonin hypothesis. This study suggests that it is more plausible that the effectiveness of depression treatment is linked to the regulation of stress in the brain. The hypothesis is that sustained stress and pain is the mechanism for the development of depression. Tailoring treatment for each individual based on his or her own conditions may add some benefit.

Many studies have shown that patients’ physical health is closely related to their mental health. People with chronic diseases such as cancer, heart disease, obesity, and diabetes are at high risk of depression. Meanwhile, people with depression are also at high risk of these chronic diseases [61]. Emotional impairment is often a reaction to organic illness, while depression can worsen comorbidities and increase mortality [62]. In ordinary psychiatric practice, psychiatrists may overlook the physical health of their patients. To address this issue, the World Psychiatric Association (WPA) has created a working group to encourage collaboration between psychiatrists and primary care physicians [63]. It is noteworthy that patients with comorbidities have been excluded from pharmaceutical clinical trials [64]. Medications may not be effective for a number of patients with a given diagnosis or comorbidities. Our study aimed to address this issue by investigating patients’ medical history (i.e., diagnoses and prior medications) that affected remission.

Some data supported guidelines for the two-tiered prescription of antidepressants [6,65,66]. For example, if patients had experienced remission in their past antidepressants, they were likely to experience remission again no matter which of the five common antidepressants they used. Patients who had tried four or more antidepressants in the prior year, i.e., treatment-resistant patients, did not benefit from escitalopram or fluoxetine, which are generally known as tier 1 medications.

The stress mechanism is also supported by the large number of diagnoses that affect response to antidepressants, as listed in Table 2 and Table 3. There were 237 unique diagnoses that affected the selection of an antidepressant, too many to discuss in this paper. The full details of these relationships are provided in the Appendices; here, we mention a few of the findings. Patients with morbid obesity benefitted from escitalopram. Patients with multiple sclerosis benefited from fluoxetine. Patients with a history of myopia benefitted from bupropion. Patients with obstructive sleep apnea benefited most from escitalopram. Depressed patients in postpartum follow-up or with normal pregnancy were likely to experience symptom remission if they were prescribed citalopram.

## Conclusions

This study aims to address the mixed findings regarding the serotonin hypothesis and the overreliance on serotonin in treating depression by providing a novel approach. The response to treatment is affected by the comorbidity of patients and their treatment. Since many comorbidities affect the response to treatment, it is unlikely that the mechanism of action of antidepressants is solely through the serotonin hypothesis. It is more likely that the response to depression treatment is through the regulation of stress in the brain. The selection of treatments is based on a patient’s medical history, and the underlying mechanisms of their depression can provide insights into novel treatment techniques. Our findings open up a wide variety of courses of action for the clinical treatment of depression, each addressing a different source of chronic stress in the brain.

## Appendices

The complete list of robust predictors of remission for the top 5 most common antidepressants is shown in Table 4.

**Table 4:** Complete list of robust predictors of remission for the top 5 most common antidepressants CPT4: Current Procedural Terminology version 4, HCl: hydrochloride, ICD9: International Classification of Diseases, Ninth Revision

Predictor	Bupropion	Citalopram	Escitalopram	Fluoxetine	Sertraline
Intercept	-3.12	-0.04	-1.66	-0.1	0.04
CPT4 Family psychotherapy (conjoint psychotherapy) (with patient present), 50 minutes		-0.17		-0.09	-0.14
CPT4 Individual psychotherapy, insight oriented, behavior modifying and/or supportive, in an office or outpatient facility, approximately 20-30 minutes	0.18	-0.57	-0.25	-0.53	-0.58
CPT4 Individual psychotherapy, insight oriented, behavior modifying and/or supportive, in an office or outpatient facility, approximately 45-50 minutes	0.09	-0.6	-0.31	-0.545	-0.615
CPT4 Pharmacologic management, including prescription, use, and review of medication with no more than minimal medical psychotherapy	0.33	-0.6	-0.19	-0.58	-0.6
CPT4 Psychiatric diagnostic evaluation		-0.28	-0.18	-0.28	-0.3
CPT4 Psychiatric diagnostic evaluation with medical services		-0.26	-0.16	-0.19	-0.17
CPT4 Psychiatric diagnostic interview examination	0.13	-0.43	-0.23	-0.38	-0.47
CPT4 Psychotherapy, 30 minutes with patient			-0.18	-0.21	-0.27
CPT4 Psychotherapy, 30 minutes with patient when performed with an evaluation and management service		-0.72	-0.37	-0.58	-0.61
CPT4 Psychotherapy, 45 minutes with patient		-0.64	-0.37	-0.55	-0.63
CPT4 Psychotherapy, 45 minutes with patient when performed with an evaluation and management service			-0.43	-0.64	-0.79
CPT4 Psychotherapy, 60 minutes with patient		-0.28	-0.19	-0.28	-0.35
Age: 13-19		-0.53	-0.19	-0.54	-0.48
Age: 20-40	-0.1	-0.17	-0.08	-0.18	-0.16
Age: 65-79			-0.13	0.1	0.03
Age: 80-89		-0.35	-0.43	-0.24	-0.24
Gender: female	-0.07		-0.02	0.04	0.03
Acetaminophen with codeine		-0.04			-0.06
Albuterol sulfate			0.06		
Allopurinol		0.06		0.12	0.09
Alprazolam		-0.07	0.03	-0.06	-0.08
Amitriptyline HCl		-0.08	0.12		-0.06
Amlodipine besylate		-0.04	-0.07		-0.03
Aripiprazole		-0.66	-0.41	-0.58	-0.73
Atenolol		0.04			
Atorvastatin calcium		0.1	0.07	0.12	0.12
Azithromycin		-0.02			-0.04
Bupropion HCl	0.44	-0.03	0.11	-0.03	
Buspirone HCl			0.14		
Celecoxib				0.05	0.05
Cetirizine HCl		0.06	-0.1		0.06
Citalopram hydrobromide		0.73	0.45	0.15	0.14
Clindamycin HCl		-0.04			
Clonazepam	0.11	-0.08	0.1	-0.07	-0.08
Clotrimazole/betamethasone dip		-0.08			-0.06
Desloratadine			-0.16		
Desogestrel-ethinyl estradiol					0.11
Dextroamphetamine/amphetamine		-0.08	0.08	-0.07	-0.07
Diazepam		-0.06		-0.07	-0.07
Divalproex sodium					-0.05
Duloxetine HCl		0.22	0.28	0.16	0.19
Escitalopram oxalate		0.55	0.94	0.2	0.19
Esomeprazole magnesium					0.04
Estradiol		0.04			0.04
Estrogen,con/m-progest acet		0.14		0.15	0.15
Estrogens, conjugated		0.07		0.09	0.08
Ethinyl estradiol/drospirenone		0.08			0.08
Ezetimibe/simvastatin		0.17		0.13	0.09
Famotidine			-0.16		
Fenofibrate		0.11		0.12	0.18
Fenofibrate, nanocrystallized		0.09	0.16		
Fenofibrate, micronized					0.09
Fentanyl			0.13		
Fexofenadine/pseudoephedrine		0.11			
Fluoxetine HCl		0.2	0.23	0.66	0.18
Fluticasone propion/salmeterol					0.04
Gabapentin			0.04		
Gemfibrozil		0.12			0.09
Hydralazine HCl		-0.12			-0.13
Hydrocodone/acetaminophen			0.05	0.02	0.02
Hydroxychloroquine sulfate					0.12
Ibuprofen		-0.06			-0.07
Ketoconazole					-0.07
Lactulose		-0.15	-0.16		
Lamotrigine		-1.23	-0.72	-1.02	-1.14
Lansoprazole		0.06			
Levonorgestrel-ethin estradiol		0.1			0.1
Levothyroxine sodium		0.07	0.06	0.06	0.08
Lisinopril				0.04	0.03
Lisinopril/hydrochlorothiazide		0.05		0.05	0.05
Lithium carbonate			-0.41	-0.77	
Loratadine		0.13			0.08
Lorazepam	0.11	-0.07		-0.07	-0.07
Lovastatin		0.11		0.08	
Meclizine HCl		-0.08			-0.09
Metformin HCl		0.04		0.04	0.07
Methotrexate sodium					0.08
Methylphenidate HCl	0.16				
Metronidazole		-0.06		-0.05	-0.07
Mirtazapine		-0.09		-0.04	-0.08
Modafinil	0.23				
Montelukast sodium					0.03
Mupirocin		-0.06			
Naproxen			-0.05		-0.06
Norethindrone					0.13
Norethindrone-ethinyl estradiol					0.08
Norgestimate-ethinyl estradiol		0.08			0.06
Omeprazole		0.03		0.06	
Oxybutynin chloride		0.06		0.09	
Oxycodone HCl			0.11		
Oxycodone HCl/acetaminophen			0.05		
Paroxetine HCl		0.2	0.22	0.12	0.18
Pravastatin sodium		0.08		0.06	0.08
Pregabalin			0.08		
Propoxyphene nap/acetaminophen			-0.07		
Quetiapine fumarate			0.09		
Rabeprazole sodium					0.07
Ramipril					0.12
Risperidone		-0.96	-0.47	-0.71	-0.98
Rizatriptan benzoate					0.07
Rofecoxib			-0.14		
Ropinirole HCl		-0.22	-0.14	-0.27	-0.29
Rosuvastatin calcium		0.08	0.06	0.08	0.09
Sertraline HCl	-0.1	0.13	0.16	0.14	0.73
Sildenafil citrate		-0.13		-0.11	-0.14
Simvastatin		0.11	0.07	0.11	0.12
Sulfamethoxazole/trimethoprim		-0.03			
Sumatriptan succinate				0.07	0.06
Terconazole		-0.1		-0.09	
Topiramate			0.1	0.05	
Trazodone HCl	0.21	-0.2		-0.21	-0.16
Triamterene/hydrochlorothiazid		0.05		0.05	
Varenicline tartrate		0.12			
Venlafaxine HCl		0.2	0.24	0.13	0.2
Warfarin sodium		0.05			
Zolpitartrate			0.05	-0.04	
ICD9 Abdominal pain, epigastric		-0.06			
ICD9 Abdominal pain, other specified site		-0.04			-0.05
ICD9 Abdominal pain, unspecified site		-0.05	-0.04	-0.04	-0.06
ICD9 Abdominal weight gain			0.06		0.06
ICD9 Absence of menstruation		-0.06	-0.1		
ICD9 Actinic keratosis					0.06
ICD9 Acute gastritis without mention of hemorrhage		-0.1			
ICD9 Acute sinusitis, unspecified					0.04
ICD9 Acute upper respiratory infections of unspecified site		-0.02		-0.04	
ICD9 Adjustment disorder with anxiety		-0.11	-0.12	-0.09	-0.16
ICD9 Adjustment disorder with depressed mood		-0.11	-0.11	-0.1	-0.09
ICD9 Adjustment disorder with mixed anxiety and depressed mood	-0.16	-0.19	-0.18	-0.18	-0.19
ICD9 Allergy, unspecified not elsewhere classified		-0.08			
ICD9 Anorexia		-0.11			
ICD9 Anxiety state, unspecified	-0.08	-0.04		-0.04	-0.03
ICD9 Atherosclerosis of native arteries of the extremities, unspecified		-0.12			-0.1
ICD9 Attention deficit disorder of childhood with hyperactivity					-0.07
ICD9 Benign hypertensive heart disease without heart failure		-0.1			
ICD9 Bipolar disorder, unspecified		-0.23		-0.25	-0.21
ICD9 Blepharitis, unspecified		-0.09			
ICD9 Body mass index between 19 and 24, adult		-0.11			
ICD9 Chest pain, other		-0.04	-0.05	-0.05	-0.06
ICD9 Chest pain, unspecified		-0.06	-0.07	-0.05	-0.07
ICD9 Chronic airway obstruction, not elsewhere classified	-0.19				
ICD9 Chronic fatigue syndrome					-0.07
ICD9 Contact with or exposure to venereal diseases		-0.15		-0.12	-0.19
ICD9 Depressive disorder, not elsewhere classified		0.1	0.08	0.09	0.085
ICD9 Dermatophytosis of nail		-0.11	-0.11	-0.1	-0.06
ICD9 Diabetes with other specified manifestations, type II or unspecified type, not stated as uncontrolled					-0.09
ICD9 Diaphragmatic hernia without mention of obstruction or gangrene		0.08			
ICD9 Diffuse cystic mastopathy		-0.06		-0.07	
ICD9 Disturbance of skin sensation		-0.04			-0.03
ICD9 Diverticulosis of the colon (without mention of hemorrhage)					0.05
ICD9 Dizziness and giddiness		-0.05	-0.06	-0.07	-0.06
ICD9 Dyspepsia and other specified disorders of the function of the stomach				-0.07	-0.06
ICD9 Dysthymic disorder		0.03	0.1	-0.05	-0.05
ICD9 Elderly multigravida, with antepartum condition or complication					0.12
ICD9 Encounter for long-term (current) use of other medications		0.05	0.07	0.05	0.04
ICD9 Encounter for other specified antenatal screening					0.1
ICD9 Encounter for routine screening for malformation using ultrasonics					0.1
ICD9 Essential hypertension, benign		0.04		0.04	0.05
ICD9 Examination of eyes and vision		0.06			
ICD9 Fitting and adjustment of vascular catheter					0.12
ICD9 Flatulence, eructation, and gas pain			-0.08	-0.06	-0.08
ICD9 Generalized anxiety disorder		-0.05		-0.08	-0.07
ICD9 Gynecological examination		0.13	-0.21		0.09
ICD9 Headache		-0.04		-0.05	-0.05
ICD9 Hypersomnia with sleep apnea, unspecified					0.06
ICD9 Hyposmolality and/or hyponatremia					-0.07
ICD9 Impotence of organic origin					-0.09
ICD9 Insomnia, unspecified		-0.03			-0.03
ICD9 Internal hemorrhoids without mention of complication					0.05
ICD9 Irregular menstrual cycle		-0.08			-0.05
ICD9 Laboratory examination			-0.06		
ICD9 Laboratory examination, unspecified					-0.07
ICD9 Leiomyoma of the uterus, unspecified				-0.1	-0.08
ICD9 Leukorrhea, not specified as infective		-0.07			
ICD9 Loss of weight		-0.11		-0.1	-0.09
ICD9 Major depressive disorder, recurrent episode, in full remission	2.08				
ICD9 Major depressive disorder, recurrent episode, in partial or unspecified remission	2.14	1.31	1.51	1.37	1.31
ICD9 Major depressive disorder, recurrent episode, mild			0.12		
ICD9 Major depressive disorder, recurrent episode, moderate		-0.15		-0.14	-0.14
ICD9 Major depressive disorder, recurrent episode, severe, without mention of psychotic behavior		-0.18		-0.15	-0.18
ICD9 Major depressive disorder, recurrent episode, unspecified	2.02	0.9	1.16	0.97	0.87
ICD9 Major depressive disorder, single episode, moderate	0.15	-0.08		-0.06	-0.12
ICD9 Major depressive disorder, single episode, severe, without mention of psychotic behavior		-0.17	-0.1	-0.12	-0.17
ICD9 Major depressive disorder, single episode, unspecified	0.16				
ICD9 Mastodynia			-0.08	-0.1	-0.11
ICD9 Mitral valve disorders			-0.08		
ICD9 Mixed hyperlipidemia		0.03	0.04	0.05	
ICD9 Morbid obesity			0.13	0.05	0.09
ICD9 Multiple sclerosis			0.13	0.17	0.1
ICD9 Myopia	0.15	0.09		0.08	0.08
ICD9 Nausea with vomiting					-0.04
ICD9 Need for prophylactic vaccination against Streptococcus pneumoniae (pneumococcus)		0.05			
ICD9 Nonallopathic lesion of the cervical region, not elsewhere classified					0.05
ICD9 Nonallopathic lesion of the lumbar region, not elsewhere classified					0.03
ICD9 Nondependent alcohol abuse, unspecified drunkenness		-0.09		-0.12	
ICD9 Nondependent tobacco use disorder	-0.32	-0.03	0.04	-0.04	-0.05
ICD9 Obesity, unspecified				0.04	0.06
ICD9 Obsessive-compulsive disorders				-0.06	-0.07
ICD9 Obstructive sleep apnea (adult) (pediatric)		0.15	0.17	0.07	0.11
ICD9 Other and unspecified alcohol dependence, unspecified drunkenness		-0.11			-0.13
ICD9 Other and unspecified bipolar disorders	-0.33	-0.26	-0.21	-0.2	-0.29
ICD9 Other and unspecified hyperlipidemia		0.04		0.04	0.04
ICD9 Other convulsions		-0.07			
ICD9 Other general counseling and advice for contraceptive management		-0.06		-0.07	
ICD9 Other malaise and fatigue	-0.07				
ICD9 Other problems related to lifestyle	-0.23			-0.09	
ICD9 Other screening mammogram		0.04			0.05
ICD9 Other seborrheic keratosis		0.05			0.04
ICD9 Other specified noninflammatory disorders of the vagina		-0.11			
ICD9 Other specified preoperative examination		0.08			
ICD9 Other testicular hypofunction			0.09		
ICD9 Overweight					-0.08
ICD9 Pain in joint, multiple sites					-0.06
ICD9 Pain in soft tissues of the limb		-0.03			-0.03
ICD9 Palpitations		-0.09	-0.09	-0.09	-0.07
ICD9 Panic disorder without agoraphobia		-0.08		-0.08	-0.06
ICD9 Paralysis agitans		-0.11			
ICD9 Personal history of tobacco use, presenting hazards to health					0.04
ICD9 Posttraumatic stress disorder		-0.19		-0.16	-0.22
ICD9 Premenstrual tension syndromes			-0.22	-0.41	
ICD9 Presbyopia		0.03			
ICD9 Presenile dementia, uncomplicated		-0.19	-0.23		
ICD9 Primary open-angle glaucoma					-0.08
ICD9 Pure hypercholesterolemia				0.02	0.03
ICD9 Rash and other nonspecific skin eruption					-0.04
ICD9 Routine general medical examination at a healthcare facility					0.02
ICD9 Routine gynecological examination	-0.04		-0.03	-0.05	
ICD9 Routine infant or child health check				-0.08	-0.04
ICD9 Routine postpartum follow-up		0.15			
ICD9 Scar condition and fibrosis of the skin			-0.08		
ICD9 Screening examination for venereal disease		-0.12	-0.05		-0.09
ICD9 Screening for malignant neoplasm of the cervix		0.05			0.04
ICD9 Screening for an unspecified condition		-0.1			
ICD9 Screening of streptococcus b					0.1
ICD9 Second-degree perineal laceration, with delivery					0.08
ICD9 Shortness of breath		-0.04			
ICD9 Special screening for malignant neoplasm of the prostate		0.07			0.07
ICD9 Suicidal ideation		-0.24			-0.13
ICD9 Supervision of other normal pregnancy		0.12		0.12	0.1
ICD9 Surveillance of previously prescribed contraceptive pill					0.08
ICD9 Unspecified adjustment reaction		-0.08	-0.09		-0.09
ICD9 Unspecified anemia		-0.05	-0.05		-0.05
ICD9 Unspecified arthropathy, site unspecified					-0.06
ICD9 Unspecified backache		-0.04			-0.04
ICD9 Unspecified constipation		-0.08	-0.07	-0.07	-0.08
ICD9 Unspecified epilepsy without mention of intractable epilepsy					-0.09
ICD9 Unspecified gastritis and gastroduodenitis without mention of hemorrhage					-0.06
ICD9 Unspecified hearing loss		-0.1			
ICD9 Unspecified hemorrhoids without mention of complication					-0.07
ICD9 Unspecified myalgia and myositis		-0.04			
ICD9 Unspecified osteoporosis		-0.04		-0.06	-0.04
ICD9 Unspecified otalgia					-0.05
ICD9 Unspecified peripheral vascular disease			-0.1		
ICD9 Unspecified seborrheic dermatitis		-0.09			
ICD9 Unspecified symptom associated with female genital organs		-0.07		-0.06	-0.09
ICD9 Unspecified tachycardia					-0.07
ICD9 Unspecified tear film insufficiency		-0.08		-0.08	-0.05
ICD9 Unspecified thrombocytopenia		-0.09			
ICD9 Unspecified vaginitis and vulvovaginitis		-0.12	-0.09	-0.09	-0.11
ICD9 Unspecified venous (peripheral) insufficiency					-0.08
ICD9 Unspecified viral infection, in conditions classified elsewhere and of unspecified site					-0.07
ICD9 Unspecified vitamin D deficiency		-0.04			-0.07
ICD9 Urge incontinence					0.07
ICD9 Urinary tract infection, site not specified		-0.04	-0.06	-0.05	-0.07
ICD9 vomiting alone		-0.07		-0.07	
Last antidepressant different and no remission	-0.54	-0.18	-0.26	-0.19	-0.19
Last antidepressant different and remission	0.77	0.37	0.61	0.41	0.36
Last antidepressant same and no remission	-0.84	-0.91	-0.99	-0.9	-0.92
Last antidepressant same and remission	1.91	0.29	1.33	0.24	0.21
Number of previous antidepressants: 2, 3			0.09		
Number of previous antidepressants: 4+			-0.27	-0.15	
Number of previous episodes: 2+	-0.11	-0.2	-0.16	-0.16	-0.18
Number of previous remissions: 2+	0.44	0.4	0.52	0.44	0.47
